# Entropy-driven machine learning for deciphering mRNA rearrangement by splicing in complex microorganisms

**DOI:** 10.3389/fbinf.2026.1889469

**Published:** 2026-09-17

**Authors:** Alessio Mancini, Emanuela Merelli, Marco Piangerelli, Sandra Pucciarelli, Leonardo Vito

**Affiliations:** 1 School of Biosciences and Veterinary Medicine, Biosciences and Biotechnology Division, University of Camerino, Camerino, Italy; 2 Clinical Laboratory Unit, Senigallia Hospital, Azienda Sanitaria Territoriale (AST), Ancona, Italy; 3 School of Science and Technology, Computer Science Division, University of Camerino, Camerino, Italy; 4 R&D Division, Vici & C. S.p.A., Santarcangelo di Romagna (RN), Italy; 5 R&D Division, Logical System Srl, Jesi (AN), Italy

**Keywords:** cis-regulatory elements, explainable machine learning, intron retention, Lempel–Ziv (LZ) measure, multiple mRNA variants, Shannon entropy, *Tetrahymena*

## Abstract

**Introduction:**

Alternative splicing allows a single gene to produce multiple messenger RNA (mRNA) variants by differential RNA processing, resulting in the translation of distinct protein isoforms. Intron retention (IR) is a specific type of alternative splicing in which introns remain unspliced in the mature mRNA. The exact regulatory code behind intron splicing remains not fully deciphered, and unraveling these mechanisms is critical to uncovering the foundations of genetic disorders: a substantial fraction of disease-causing mutations disrupt intron splicing.

**Methods:**

We applied explainable machine learning (xML) models to intronic sequences from four species of the ciliate genus Tetrahymena (*T. thermophila*, *T. malaccensis*, *T. borealis* and *T. elliotti*), which allow the intron retention process to be analysed without tissue-specific confounders. Retained introns (RIs) and constitutively spliced introns (CSIs) were extracted through an automated Galaxy pipeline and described by several features of the intronic sequences, including the absence of repetitive nucleotide motifs – quantified as entropy – the GC content, the complexity of the secondary structures as estimated by the Lempel–Ziv (LZ) measure, and the effective distance between the branch point and the 3′ splice site. Four classifiers were compared by 10-fold cross-validation within the training set and evaluated on an independent test set.

**Results:**

Across 289.6 k analysed introns, the C5.0 decision tree gave the best and most consistent performance, ranking first in three of the four species (accuracy 0.948–0.984). The key distinguishing features of retained introns were a higher entropy (Shannon entropy above 1.67, versus below 1.53 for constitutively spliced introns, against a theoretical maximum of 2) and more complex secondary structures near the 3′ splice sites, together with a greater effective branch-point-to-3′-splice-site distance and a relative enrichment of the GC dinucleotide.

**Discussion:**

These features converge on weaker, less accessible splicing signals, which may impair splice-site recognition and bias the outcome towards retention. Our work identifies key sequence-level features predictive of intron retention in Tetrahymena and could provide a generalizable, explainable ML framework for investigating splicing regulation in other eukaryotes.

## 1 Introduction

Machine learning (ML) has become an increasingly powerful tool for tackling complex biological questions (Mancini et al., 2020). One of the most significant challenges in molecular biology is deciphering the gene regulation mechanisms and proteome diversity driven by mRNA rearrangement. Addressing this challenge demands the analysis of vast genomic and transcriptomic datasets, a task where machine learning can provide invaluable support.

If genomic DNA can be likened to a read-only word processing document, mRNA serves as the editable transcript, allowing modifications to shape the final output. In newly synthesized precursor messenger RNA (pre-mRNA), sequences called introns are excised, while sequences called exons are maintained in the mature mRNA. This biological mechanism is called intron splicing (Supplementary Figure S1). Intron splicing is a highly sophisticated process that varies depending on the intron type: it can occur via self-splicing or be mediated by additional RNA molecules and proteins, as seen in spliceosomal introns. Certain introns are not always removed during splicing: in some transcripts they are retained, as in Alternative Splicing (AS), which contributes to proteomic diversity. AS enables the generation of multiple mRNA variants from a single gene sequence, which are subsequently translated into distinct protein isoforms (Barash et al., 2010; Nilsen and Graveley, 2010).

Most of the information necessary for splicing reside in intronic cis-regulatory elements. In both spliceosomal and self-splicing mechanisms, splice sites (SS) are characterized by highly conserved motifs: GU and AG dinucleotides at the 5′ and 3′ ends, respectively. The adenine (A) residue within the degenerate branch point (BP) motif, 5′YNYYRAY3′, triggers the splicing reaction. The 5′ splice site is termed the “donor” because it donates the phosphodiester bond connecting it to the upstream exon during the first transesterification reaction. In this reaction, the 2′-OH group of the branch point adenosine attacks the phosphodiester bond at the 5′SS. Conversely, the 3′ splice site (3′SS) is referred to as the “acceptor” because it accepts the second transesterification reaction, when the 3′-OH group of the upstream exon attacks the phosphodiester bond at the 3′SS (Lee and Rio, 2015).

AS provides an explanation for how the approximately 20,000 protein-coding genes in the human genome generate the vast diversity of proteins, cell types, and tissues (Ezkurdia et al., 2014). Intron retention (IR) was considered a prevalent form of alternative splicing in unicellular eukaryotes (Sebé-Pedrós et al., 2013). Recent RNA-seq data from over forty diverse human and mouse cell and tissue types have revealed that IR is significantly more common in mammals than previously recognized (Braunschweig et al., 2014). Evidence suggests that retained introns play a widespread role in functionally tuning transcriptomes by altering the expression of relatively low-abundance transcripts (Monteuuis et al., 2019).

Efforts to decipher the splicing code have involved extensive analyses of RNA features such as exon length and secondary structure (Barash et al., 2010), or by applying integrated Bayesian models (Zhang et al., 2010). In this study, explainable machine learning (xML) models were applied to investigate the mechanism underlying IR. We developed an interpretable machine learning model that analysed vast amounts of genomic and transcriptomic data to identify key features in the primary and secondary structures of pre-mRNA that distinguish retained introns (RIs) from constitutively spliced introns (CSIs). Intronic sequences from species within the ciliate genus *Tetrahymena* were analysed (Piersanti et al., 2021; Mancini et al., 2018). *Tetrahymena* provides an ideal case study for IR, as gene expression and pre-mRNA processing are not biased by tissue type. Previous transcriptomic work on *T. thermophila* characterised IR as the dominant alternative-splicing event and identified intron-definition as the prevalent splice-site recognition mode (Xiong et al., 2012), while Csűrös et al. (2016) showed that ciliate introns follow a bimodal length distribution corresponding to two splice-site recognition modes; both findings provide essential biological context for the present analysis.

## 2 Methods

The analysis pipeline was structured into five key phases (from A to E in Supplementary Figure S2): Conceptualization, Data Acquisition, Data Preparation, Machine Learning Modeling, and Results Interpretation.

### 2.1 Conceptualization

(A). The primary objective of this phase was to design a strategy for identifying sequence-specific intron features that influence intron retention. To achieve this goal, we prioritized the use of machine learning methodologies, specifically supervised classification techniques, to differentiate between Retained Introns (RIs) and Constitutively Spliced Introns (CSIs). The approach was further refined by integrating xML methods, which improved model interpretability and enabled the identification of the most relevant classification features.

### 2.2 Data acquisition

(B). Genome and transcriptome data from *Tetrahymena* used in this study were obtained from the *Tetrahymena* Genome Database (https://tet.ciliate.org) for genome assemblies, and from the European Nucleotide Archive at the European Bioinformatics Institute for transcriptomic reads (https://www.ebi.ac.uk/). For genome-wide mapping, a minimum of four transcriptomes for each *Tetrahymena* species (some of which obtained from different cellular states, e.g., starvation, conjugation, growth) were used (Supplementary Table S1). RNA STAR v2.4.2a (Dobin et al., 2013) was used for reads mapping: only those with at least 85% of alignment were used for the next steps.

### 2.3 Data preparation

(C). Raw reads were preprocessed using Trimmomatic v0.32 (Bolger et al., 2014) to remove low-quality sequences and adapter contamination. This tool supports both Paired-end and Single-end modes, utilizing paired read information to precisely detect adapter fragments or PCR primers. FASTQ files were processed using Phred with +33 or +64 quality scoring systems. For this analysis, the trimming parameters included adapter removal, a sliding window approach with a Phred score threshold of 30 (indicating 99.9% base-calling accuracy), and truncation of reads to a minimum length of 45 bp.

For each BAM file, transcriptomes were assembled independently using Cufflinks v2.2.1 (Trapnell et al., 2012; Supplementary Figure S2). Given that ciliate introns are shorter than those from mammals (ranging from 40 to 150 bp), the -I parameter was reduced from the default 300,000 bp to 4,000 bp, well above the 40–150 bp typical range, to constrain the search space to plausibly intronic distances. The -u parameter was also employed to prioritize high-ranking alignments for multi-mapped reads. Cufflinks reconstruct transcripts from RNA-seq reads aligned to the genome, aiming to accurately infer splicing structures while accounting for potential alternative splicing events. To produce a parsimonious transcriptome assembly, Cufflinks reports only the minimal set of full-length transcript fragments (transfrags) necessary to explain observed splicing patterns. Additionally, it quantifies the expression of each transfrag while filtering out low-abundance and artefactual fragments, such as immature transcripts, ensuring a high-quality assembly for downstream analysis. Cuffmerge was utilized to remove the redundant isoforms in different samples, functioning as a meta-assembler. Similar to how Cufflinks processes reads, Cuffmerge merges assembled transfrags from multiple samples in a parsimonious manner. When a reference genome annotation is available, Cuffmerge integrates reference transcripts into the merged assembly through a reference annotation-based transcript (RABT) assembly, producing a single complete annotation file for downstream differential analysis. In cases where genes exhibit low expression, sequencing depth may be insufficient for full reconstruction in individual replicates. Yet merging replicate assemblies with Cuffmerge often facilitates the recovery of complete gene structures. This process also integrates newly discovered isoforms with known ones, yielding more complete gene models. Once sample assemblies are merged, the final consolidated assembly serves as a foundation for identifying genes and transcripts that are differentially expressed or regulated across experimental conditions.

To perform RNA-seq-based IR detection (Supplementary Figure S2), iREAD (Li et al., 2020) and ASTALAVISTA (Foissac and Sammeth, 2007) were selected as tools. Branching points were identified by using SVM-BPfinder (Corvelo et al., 2010) and BranchPointer (Signal et al., 2018). The two tools were combined via a consensus strategy, retaining only branch point positions called by both predictors; in cases of disagreement, the prediction with the higher confidence score was used.

### 2.4 Scalable bioinformatics workflows

To facilitate the analysis, bioinformatics tools and custom scripts were integrated within the Galaxy platform (Afgan et al., 2018) (Supplementary Figure S2, phase C). The pipeline consists of a series of stages that automate the RIs and CSIs extraction process: identifying intron sequences from GTF coordinates, selecting RIs sequences based on iREAD coordinates, excluding RIs to isolate CSIs, removing outlier sequences (i.e., introns longer than 1,000 bp or shorter than 20 bp, a post-assembly filter tighter than the Cufflinks -I limit of 4,000 bp, intended to discard putatively chimeric or unprocessed transcripts that survived the more permissive assembly step), and performing secondary structure analysis using RNAfold (Lorenz et al., 2011). Automating the extraction process of RIs and CSIs makes it reproducible and consistent across species.

### 2.5 Feature engineering

To facilitate machine learning, intronic sequences from the CSIs and RIs datasets were processed to capture both the statistical complexity and the biophysical characteristics of the sequences. Features such as Shannon entropy, dinucleotide pair frequencies (six unordered pair probabilities; Karlin and Burge, 1995; see Supplementary §1.1.1), Lempel–Ziv complexity (Gusev et al., 1999; Orlov and Potapov, 2004), and secondary-structure properties (Lempel–Ziv complexity on the dot-bracket secondary structure, average accessibility, and effective branch point–to–3′ splice-site distance), together with MaxEntScan (Yeo and Burge, 2004)-derived 5′ and 3′ splice-site strength scores were selected to provide a multidimensional representation of the intronic sequences, enabling the development of predictive models that integrate both sequence-level and structural-level information. A short description of each feature is provided in A short description of each feature is provided in Supplementary Material (see also Supplementary Figure S3 and Supplementary Table S4).

### 2.6 Modeling

(D). It was based on the Machine Learning (ML) pipeline (Supplementary Figure S4). It consists of data visualization, pre-processing, data splitting, modeling, and model evaluation main steps. In the pre-processing phase, we utilized Principal Component Analysis (PCA) for dimensionality reduction to eliminate highly correlated features and mitigate overfitting. The distribution of RIs and CSIs classes within the dataset exhibits a marked imbalance reflecting the inherent characteristics of intron retention. Classification performance on imbalanced datasets often favors the majority class, potentially compromising the accurate classification of the minority class. To address this challenge, oversampling techniques, such as SMOTE (Chawla et al., 2002) and ROSE (Menardi and Torelli, 2014), along with a downsampling approach, were applied during the Splitting Phase to balance the dataset and enhance model performance. To avoid data leakage, introns from the same or highly similar genes were consistently assigned to the same split (75% training or 25% test). Redundant sequences were further reduced by clustering at 95% nucleotide similarity prior to model training. Within the training set of each species, 10-fold cross-validation guided the comparison and selection of the algorithms. To prevent further data leakage, correlated variable removal, PCA, and SMOTE were all fitted exclusively within each training fold. The selected models were then evaluated on the independent 25% test set, whose performance is reported in Table 1. The models included Support Vector Machine (SVM (Smola and Schölkopf, 2004)), a maximum-margin classifier; C5.0 (Pandya and Pandya, 2015), a decision tree model; Neural Networks, capable of capturing non-linear relationships; and Adaboost M1 (Freund et al., 1999), an ensemble method combining weak learners. Upon completion of cross-validation across the three balancing methods, the results indicated that the C5.0 model (Table 1), in combination with SMOTE, produced the most consistent performance across species (see Results for per-species details). We chose C5.0 for the interpretation phase given this consistency and its explicit decision rules. For classification purposes, RIs were assigned to the negative class and CSIs to the positive class. With this definition, sensitivity in Table 1 indicates the proportion of CSIs correctly identified, whereas recall for the minority RI class corresponds to specificity. In the evaluation phase, several statistical rates for classification modeling were investigated. Despite this being a long-standing issue in ML, no widespread consensus has been reached on a unified elective chosen measure yet. F1 and accuracy are reliable when applied to balanced datasets and produce misleading results when applied to imbalanced cases. The MCC criterion is intuitive: it becomes apparent that the classifier must make correct predictions about both most negative cases and most positive cases, regardless of their ratio in the overall dataset. This is evident by examining its formula:

$$\text{MCC}=\frac{TP\times TN-FP\times FN}{\sqrt{\left(TP+FP\right)\left(TP+FN\right)\left(TN+FP\right)\left(TN+FN\right)}}$$

where TP and TN are True Positive and Negative, respectively, and FP and FN are False Positive and Negative.

**TABLE 1:** Machine learning models performance evaluation on the independent 25% test set (after SMOTE balancing of the training data), using iREAD introns recognition. Under our convention, CSIs are the positive (majority) class and RIs the negative (minority); recall on the RI class therefore corresponds to specificity.

Algorithm	AUC-PR	AUC-ROC	Accuracy	Precision	F1 score	MCC	Sensitivity	Specificity
*Tetrahymena thermophila*								
Adaboost M1	0.990	0.988	0.989	0.982	0.989	0.979	0.997	0.982
NeuralNetworks	0.970	0.970	0.841	0.870	0.832	0.684	0.798	0.883
C5.0	0.990	0.980	0.961	0.938	0.987	0.959	0.922	0.942
SVM	0.990	0.990	0.953	0.975	0.957	0.917	0.939	0.974
*Tetrahymena malaccensis*								
Adaboost M1	0.930	0.940	0.920	0.943	0.918	0.850	0.880	0.961
NeuralNetworks	0.810	0.810	0.709	0.721	0.691	0.419	0.660	0.752
C5.0	0.980	0.980	0.948	0.921	0.948	0.898	0.978	0.919
SVM	0.840	0.860	0.781	0.786	0.772	0.562	0.759	0.802
*Tetrahymena borealis*								
Adaboost M1	0.860	0.870	0.784	0.748	0.765	0.479	0.727	0.732
NeuralNetworks	0.910	0.920	0.783	0.785	0.777	0.567	0.770	0.797
C5.0	0.980	0.980	0.984	0.972	0.984	0.970	0.997	0.973
SVM	0.940	0.950	0.919	0.952	0.914	0.840	0.879	0.957
*Tetrahymena elliotti*								
Adaboost M1	0.850	0.870	0.745	0.734	0.727	0.489	0.719	0.769
NeuralNetworks	0.840	0.860	0.751	0.730	0.738	0.501	0.747	0.754
C5.0	0.990	0.990	0.961	0.938	0.959	0.922	0.981	0.942
SVM	0.980	0.990	0.960	0.939	0.959	0.922	0.968	0.943

### 2.7 Results interpretation

(E). This process facilitates the interpretation of artificial intelligence models, particularly those classified as a black box, such as neural networks (Vimbi et al., 2024). The implementation of these methodologies allows for further analysis of the phenomenon under investigation. For instance, it is observed that the feature secondary-structure Lempel–Ziv complexity (SsLZ), when values are below 1.10, contributes to the classification of the intron as a CSIs. The maximum Shannon entropy for an alphabet of length 4 is H_max_(X) = log_2_(4) = 2; Shannon entropy values lower than 1.53 indicate reduced sequence disorder and contribute to classification as CSIs, whereas values greater than 1.67 indicate higher disorder and contribute to classification as RIs. In our RIs and CSIs classification experiment, multiple bioinformatics tools were utilized alongside custom scripts to complete the entire pipeline. To streamline and standardize this process, we integrated our pipeline into the Galaxy platform as shown in Supplementary Figure S5. Docker images for the pipeline can be downloaded at https://hub.docker.com/r/dockervito/galaxybiounicam4. This containerized solution ensures reproducibility and simplifies the deployment of the analysis workflow across diverse computational environments.

## 3 Results

### 3.1 Detection of the retained introns

The study was conducted on four *Tetrahymena* species: *T. thermophila, T. elliotti, T. borealis,* and *T. malaccensis* following the data pipeline described in Supplementary Figure S2 and described under Methods.

The genomes were downloaded from https://tet.ciliate.org/downloads.php.

Transcriptomic data used in this study are reported in Supplementary Table S1 and the transcriptomes statistics in Supplementary Table S2. The GC content across the analyzed species ranged from 22.6% to 24.0%, while the mean assembly length per genome ranged from 183 kb (*T. malaccensis*) to 571 kb (*T. thermophila*), with N50 values between 496 kb and 1.02 Mb (Supplementary Table S2). Read alignment rates ranged from 85.4% to 97.4%, mapped using RNA STAR. Transcriptomic data from different physiological states were collected and underwent stringent quality checks to ensure suitability for intronic sequence analysis.

Detecting IR presents several challenges, such as distinguishing genuine IR events from transcriptional noise, addressing potential DNA contamination from insufficient DNase treatment, and accounting for unprocessed pre-mRNA transcripts. We tested five tools (Supplementary Table S3), among these, iREAD and ASTALAVISTA gave the best results. These two tools were selected on the basis of their complementary detection strategies (iREAD focuses on read coverage uniformity, while ASTALAVISTA identifies AS events from splice-junction graphs) and their ease of integration into the Galaxy pipeline. iREAD is a tool specifically tailored for analyzing IR, and employs a threshold based on read coverage uniformity, quantified using Shannon entropy (lower entropy indicates non-uniform intron coverage). This read-coverage Shannon entropy used by iREAD for IR calling is mathematically independent of the nucleotide-sequence Shannon entropy used as an ML feature (§2.5; Supplementary §1.1.2); IR detection and feature extraction are therefore not circular. ASTALAVISTA uses junction positions from GTF files to identify and classify alternative splicing (AS) events, ASTALAVISTA characterizes diverse splicing types within localized gene regions by utilizing AS graphs, which go beyond the binary inclusion/exclusion analysis of individual exons or introns. Therefore, we used the data obtained from iREAD, which directly provides IR-specific intron coordinates suitable for downstream classification, while ASTALAVISTA classifies AS events at the splice-graph level (Figure 1). The intron length distribution, derived from quantiles at the specified probabilities Φ = [0.02, 0.2, 0.4, 0.6, 0.8, 0.98], revealed that 95% of RIs and CSIs fell within the 20 to 160 bp range. Overall 289.6 k introns (286.5 k CSIs vs. 3.1 k RIs) were analyzed. After balancing with SMOTE (Synthetic Minority Oversampling Technique), the dataset contained equal numbers of both classes. Introns longer than 1,000 bp or shorter than 20 bp were therefore considered outliers and removed prior to classification (see §2.4 for details).

**FIGURE 1:**
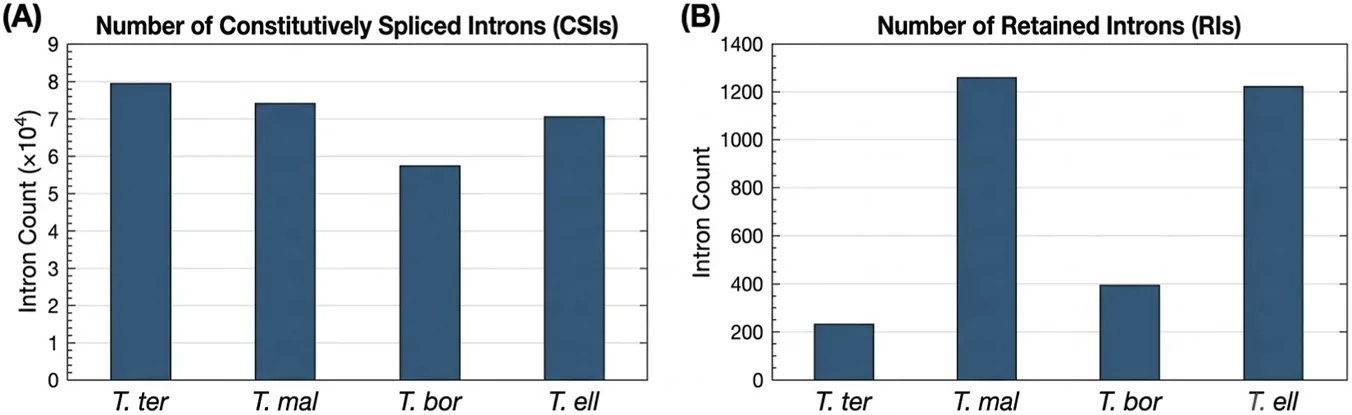
Number of constitutively spliced introns **(A)** and retained introns **(B)** as found by iREAD. T. ter: T. thermophila, T. mal: T. malaccensis; T. bor: T. borealis, and T. ell: T. elliotti.

### 3.2 Identification of intron’s features by ML-based approach

To identify the sequence characteristics most strongly associated with IR, a ML-based approach was developed to classify introns as either retained or non-retained. Using a classification model, xML techniques were applied to interpret the model’s decisions and identify specific nucleotide features that contribute to the prediction of IR. Four ML models (SVM, C5.0, Neural Networks, Adaboost) were tested using features such as entropy, LZ complexity, splice site strength.

Table 1 reports the performance evaluation of ML models for intron recognition processed with SMOTE, using iREAD introns recognition. The analysis spans the four *Tetrahymena* species*: T. thermophila, T. malaccensis, T. borealis, and T. elliotti*.

Full per-species metrics are reported in Table 1. Across the four species, C5.0 gave the best and most consistent performance, ranking first in three (*T. malaccensis*, accuracy 0.948; *T. borealis*, 0.984; *T. elliotti*, 0.961), while Adaboost M1 marginally outperformed it on *T. thermophila* (accuracy 0.989 vs. 0.961). SVM tracked C5.0 closely throughout, whereas Neural Networks were the most variable, performing well on *T. thermophila* but dropping markedly on *T. malaccensis* (accuracy 0.709, MCC 0.419) and *T. elliotti* (AUC-ROC 0.86). This inter-species variability may reflect differences in genome-assembly quality and evolutionary divergence of splicing factors. C5.0 was retained for the interpretation stage for its explicit, xML-exploitable decision-rule structure rather than its raw predictive metrics.

Since the results obtained with C5.0 appeared as the most reliable, we used this data set. The results of the explainable xML algorithm are presented in Figure 2 and in Figure 3.

**FIGURE 2:**
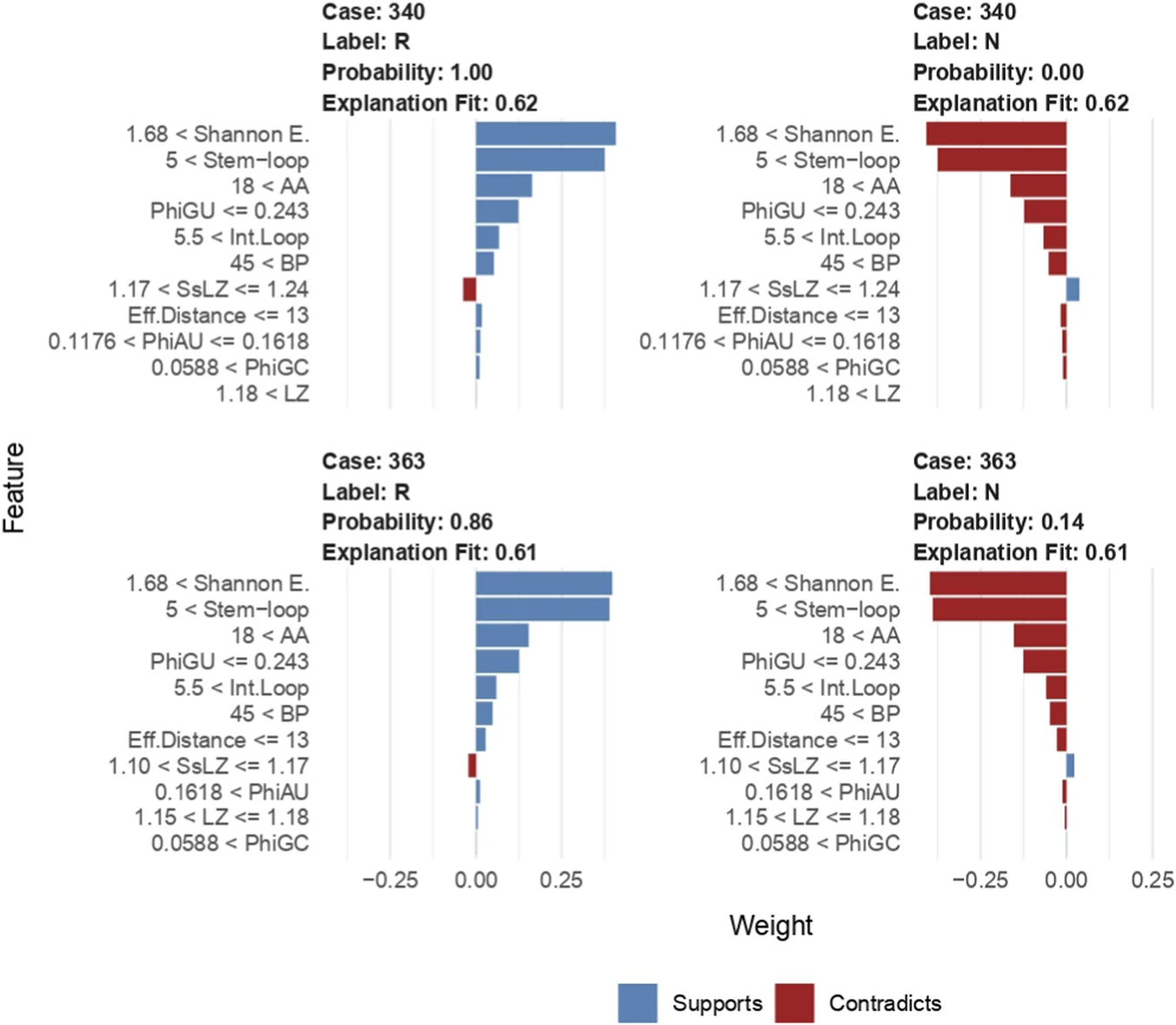
Intron’s features obtained from C5.0 data. In the figure are reported two observations, i.e., cases 340 and 363 for both retained (R) and non-retained introns (N). The individual contributions of these features are shown in blue (supporting) and red (contradicting), allowing interpretation of which aspects of the data are driving the prediction for RIs. Probability, i.e., the confidence score assigned by the model to that class, Explanation Fit, i.e., How well the explanation approximates the model’s decision (quality of interpretation). How to Read the Bars: Blue bars (Supports), Features that favor the respective class; Red bars (Contradicts), Features that oppose the respective class.

**FIGURE 3:**
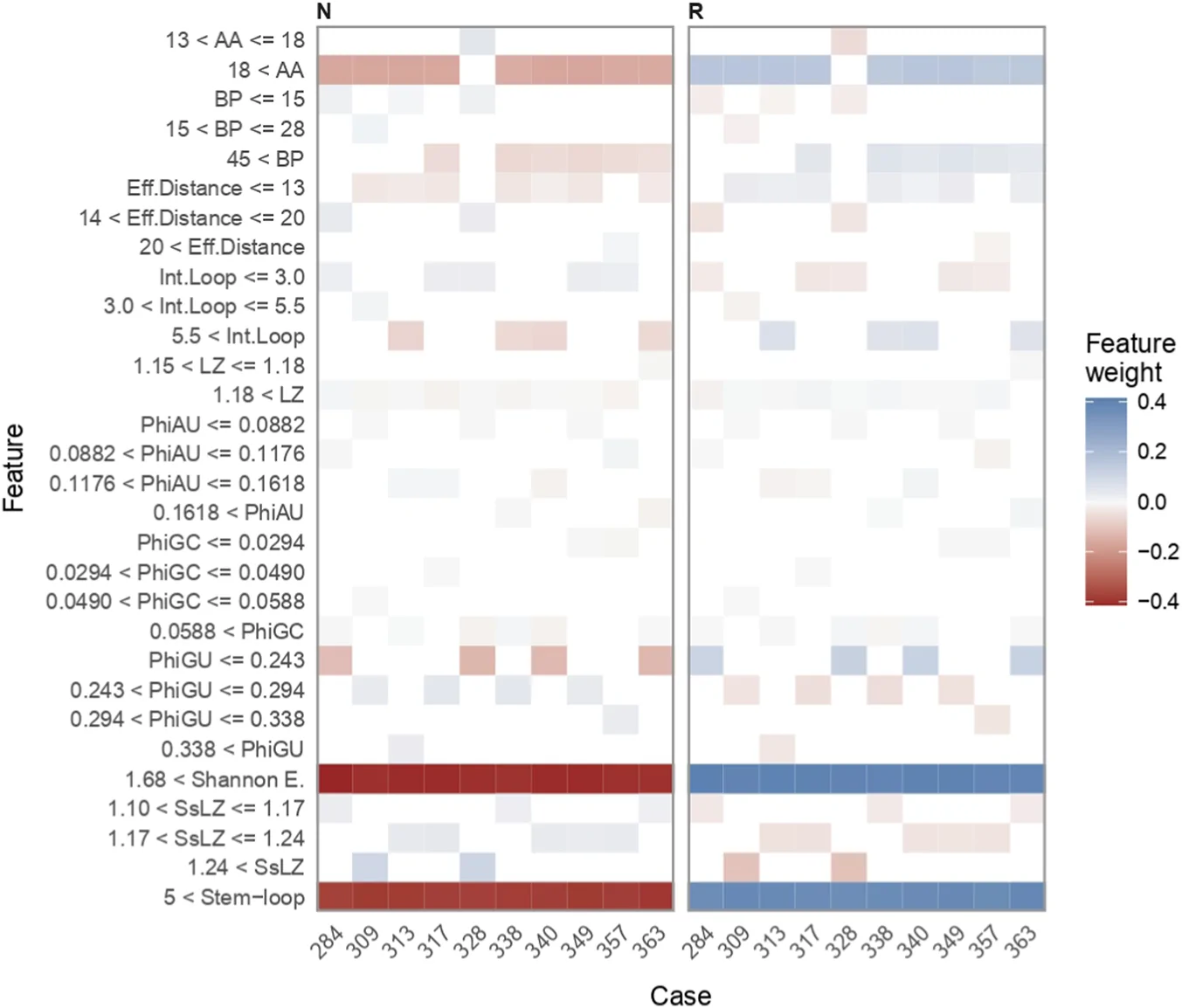
Heatmap displaying feature importance for predicting retained introns (R) in C5.0. The x-axis shows individual cases, while the y-axis lists specific features with their threshold values. Cell colors indicate feature contributions to the model’s predictions: blue for positive (supporting) and red for negative (contradicting) contributions.

The horizontal axis (Weight) indicates the strength of the effect: the longer the bar, the more influential the feature is in the model’s decision.

In general, the analysis of the sequence features highlighted significant differences between RIs sites and the CSIs sites (Figure 4). Specifically, RIs sites exhibited a higher Shannon entropy and Lempel–Ziv complexity, indicative of a greater sequence randomness (Figure 5).

**FIGURE 4:**
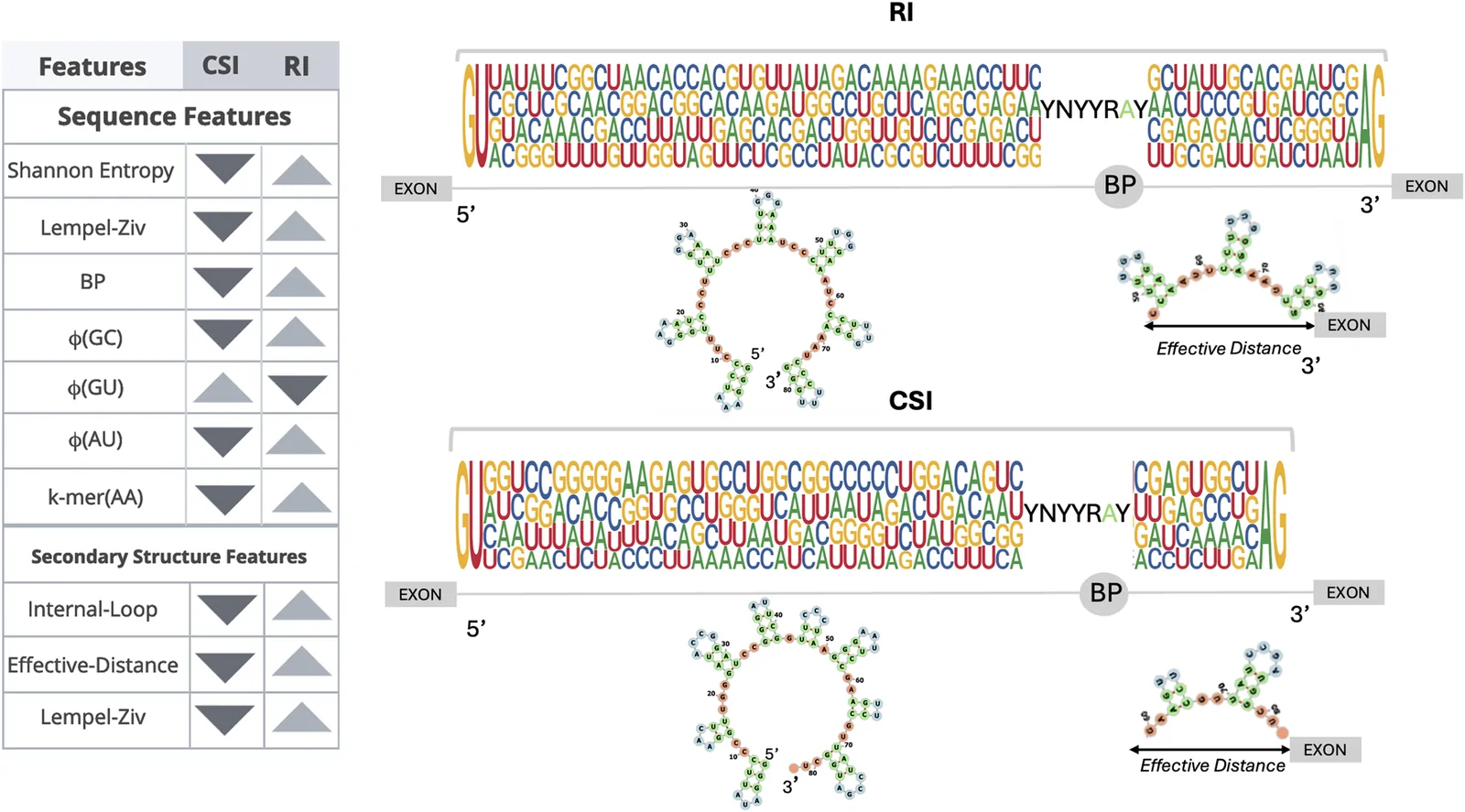
Differences in features between RIs and CSIs in C5.0. On the left, the features that distinguish RIs from CSIs are listed, with arrows indicating whether the frequency of each feature is higher or lower in the respective intron classes. On the right, a graphical representation illustrates these differences, with GU and AG denoting the highly conserved dinucleotides at the 5′ and 3′ ends of the introns, respectively. The degenerated YNYYRAY sequence, with the highly conserved A (highlighted in green), represents the branching motif. The horizontal dotted lines indicate the effective distance between the branch point and the 3′ splice site, i.e., considering the RNA secondary structures. The higher entropy in the RIs sequences is represented by letters of uniform size, indicating equal probability for each nucleotide. In contrast, the lower entropy in the CSIs sequences is represented by letters of varying sizes, reflecting the differing probabilities of each nucleotide.

**FIGURE 5:**
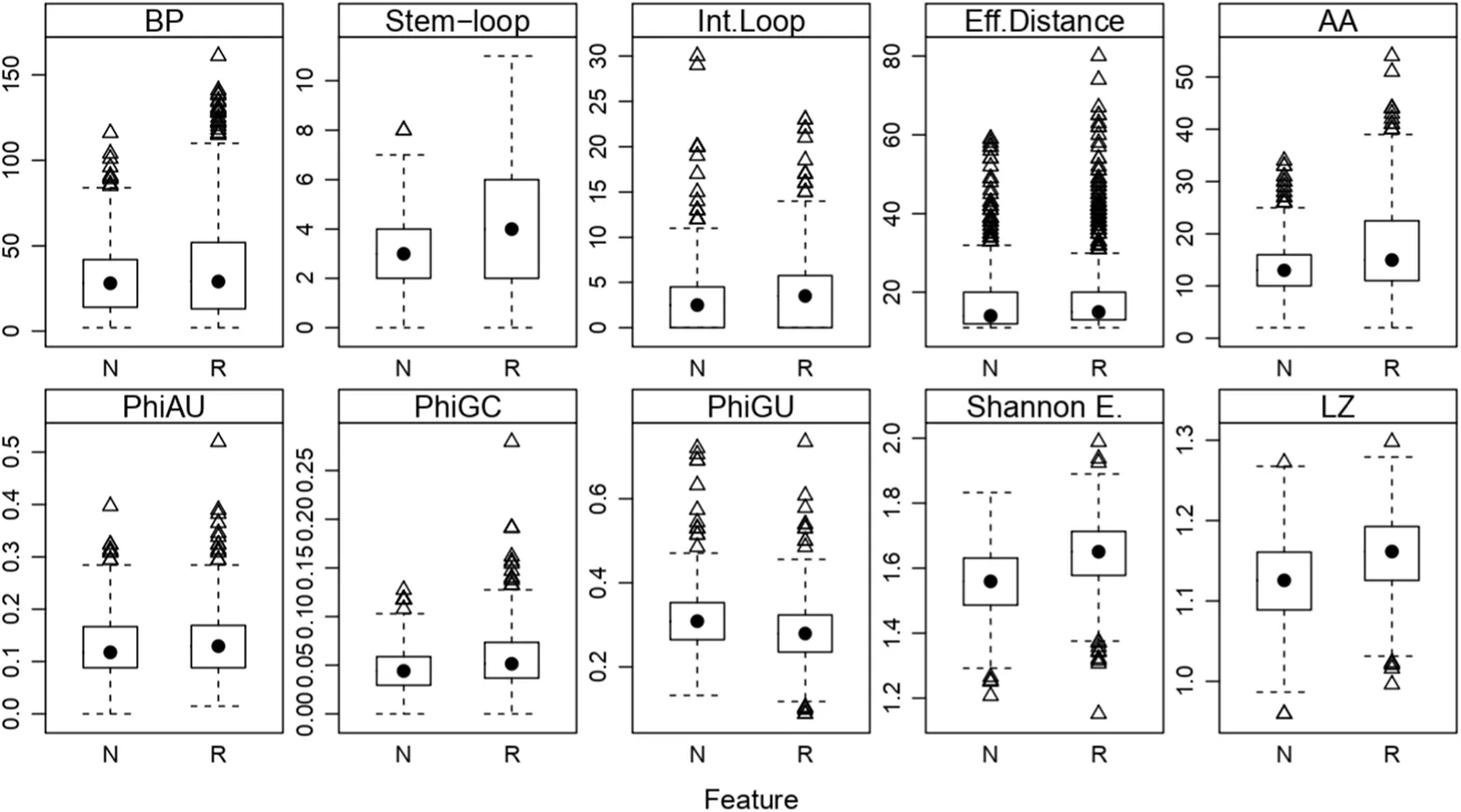
Boxplots comparing various structural and complexity metrics between RIs and CSIs of *Tetrahymena* in C5.0. The central box represents the interquartile range (IQR), with the median value indicated by a dot within the box. Features were selected after removing correlations, applying PCA, and performing Model Interpretation Analysis. The SL (Sequence Length) characteristic, due to its inherent nature and characterization, was excluded from the applied method.

These findings suggest that RI sites may have an increased sequence variability compared to CSI sites, potentially contributing to their distinct splicing behavior. In RIs, the branch point appears effectively farther from the 3′ splice site within the folded structure compared to CSIs, consistent with a slightly longer linear distance. RIs showed a tendency towards a higher median length compared to CSIs, although 95% of all introns fall within the 20 to 160 bp range, resulting in a slightly higher distance between the BP and the 3′ splice site. As a result, the structures of RIs between the BP and the 3′ splice site are inherently longer, which likely accounts for the higher number of internal loops and stems observed. A greater distance between these two elements is likely associated with introns that undergo AS (Gooding et al., 2006). The higher sequence variability of RIs is consistent with weaker, less constrained splice signals, which may favour retention and a wider range of protein isoforms. These sequence-level features are also relevant to the evolution of splicing and to cellular adaptation.

## 4 Discussion

Our xML approach identified two interpretable signatures that separate retained from constitutively spliced introns in *Tetrahymena*: higher Shannon entropy in the primary sequence and increased Lempel–Ziv complexity of the secondary structure between the branch point and the 3′ splice site. Decision-tree thresholds (SsLZ ≤1.10 for CSI, Shannon entropy >1.67 for RI) provide concrete, testable rules. Both features point to a weakening of the splicing signal: a less repetitive primary sequence offers fewer recognition motifs, while a more complex local structure increases the chance that the AG 3′SS or the BP is sequestered in a stem or loop. In mammals, recognition of the 3′ splice site can be influenced by a scanning mechanism and by the structural accessibility of the AG dinucleotide (Smith et al., 1993); our results suggest that analogous structural constraints may also weaken 3′ splice-site recognition in *Tetrahymena*. Overall, the failure to recognize the AG 3′ splice site may result from its greater effective distance from the BP within the folded structure (in line with a slightly larger linear distance, see Results) or its sequestration within a loop. These findings are consistent with previous studies showing that alternative splicing is regulated by both the strength and the competition of splicing signals (Lee and Rio, 2015). CA repeats (Hui et al., 2005; Wang and Burge, 2008) and G-rich motifs, such as G triplets (GGG) or G runs (Gn, where n ≥ 3), have been reported to enhance intron splicing by facilitating the binding of heterogeneous nuclear ribonucleoproteins (hnRNPs). However, our results indicate that these repeats are infrequently detected in RIs. In contrast, we observed a higher frequency of the GC dinucleotide, which has been associated with drug-induced IR of human introns expressed in mice (Vigevani et al., 2017). A comparable supervised-classification framework for IR vs. constitutively spliced introns has been applied to *Arabidopsis thaliana* by Mao et al. (2014), providing a plant-side benchmark for the present results. In addition, Wong et al. (Wong et al., 2018; Wong and Schmitz, 2022) suggest an “IR code” that includes high GC content, short length, weak splice sites and BPs, as well as an association between IR and a slower rate of transcription elongation, which reflects inefficient splicing factor recruitment.

To make explicit how the most discriminative features relate to splicing mechanism, we consider them in turn. (i) The higher Shannon entropy of retained introns reflects a less repetitive primary sequence and therefore a paucity of the short cis-regulatory motifs, such as CA repeats and G-rich elements (G triplets and G runs), that recruit hnRNPs and U1 snRNP to define splice sites and intron borders (McCullough and Berget, 1997; 2000; Hui et al., 2005; Wang and Burge, 2008); consistent with this, these motifs are infrequent in our RIs. (ii) The increased Lempel–Ziv complexity of the secondary structure around the 3′ region (SsLZ) indicates a more intricate local fold, which can occlude the AG dinucleotide of the 3′ splice site or the branch-point adenosine within a stem or loop and thereby impede scanning-based recognition of the 3′ splice site (Smith et al., 1993; Plass and Eyras, 2014). (iii) The greater effective branch-point–to–3′-splice-site distance in the folded structure is consistent with the association between distal branch points and alternatively spliced introns reported by Gooding et al. (2006). (iv) Finally, the relative enrichment of the GC dinucleotide in RIs mirrors its reported role in intron retention (Vigevani et al., 2017) and the high-GC component of the mammalian “IR code” (Wong et al., 2018; Wong and Schmitz, 2022). Together, these features converge on a single interpretation: retained introns carry weaker, less accessible splicing signals, which lowers the efficiency of 3′-splice-site definition and biases the outcome towards retention.

IR regulates the production of protein isoforms, RNA stability, and translation efficiency during normal development and in response to disease. It is a key mechanism for responding to environmental stresses, such as thermal, nutritional, and oxidative stress (Wong and Schmitz, 2022) and it affects genes implicated in temperature-dependent sex determination across multiple species, including fish and reptiles (Georges and Holleley, 2018). IR contributes to growth and development of plants (Jabre et al., 2019) and in yeast, the introns of ribosomal proteins can mediate inducible phenotypic heterogeneity that confers a clear fitness advantage. Intron splicing is, therefore, clearly relevant to inducible phenotypic heterogeneity, including neuronal plasticity (Furlanis and Scheiffele, 2018), and for adapting to environmental changes (Lukačišin et al., 2022). Unraveling the splicing pattern code of any primary transcript can contribute to understanding the role of IR in tuning plasticity and soft (epigenetic) inheritance mechanisms of environmental adaptation. Interestingly, among the tested algorithms, the C5.0 decision tree achieved the highest predictive performance in three out of four *Tetrahymena* species (Table 1). One possible explanation is that decision tree models are particularly well suited to capture discrete sequence rules and thresholds, such as the presence of conserved splice site motifs, the effective distance between branch point and 3′ SS, or the local enrichment of CA- and G-runs. These categorical boundaries are readily translated into branching rules in a tree structure, enhancing both performance and interpretability. Previous studies have shown that decision tree–based models can effectively model cis-regulatory logic in splicing by partitioning the sequence space into biologically meaningful rules, whereas linear models may fail to capture such non-linear interactions (Lee and Rio, 2015). Moreover, unlike neural networks, which tend to overfit on smaller datasets, decision-tree methods such as C5.0 retain robustness on moderately sized data and produce explicit, feature-level rules suitable for interpretation. Because C5.0 yields explicit, feature-level rules at comparable accuracy, it is well suited to a setting where interpretability matters as much as prediction.

A further, architecture-related factor may also contribute to the lower accuracy of the neural networks tested here. The feedforward networks we used treat the engineered features as an unordered vector and are not tailored to the sequential nature of nucleotide data; architectures designed for sequence modelling, such as bidirectional Long Short-Term Memory (biLSTM) networks, can capture positional and longer-range dependencies along the intron and have proven effective for related sequence-classification tasks. Such models might recover patterns that our feedforward networks miss, albeit at the cost of the transparent, rule-based interpretability that motivated our use of C5.0. We did not pursue this direction, as the decision-tree models already achieved high and directly interpretable performance, but it represents a promising avenue for future work.

Several aspects of these results merit further consideration. First, only ∼1% of introns in *Tetrahymena* (3.1 k RIs out of 289.6 k introns, corresponding to approximately 734 introns per Mb of assembled genome, about 14-fold higher density than the human reference, ≈53 introns/Mb) are retained, markedly lower than the IR rates reported in mammalian transcriptomes (Braunschweig et al., 2014). This contrast is consistent with the absence of tissue-specific splicing programs in this unicellular ciliate and supports the interpretation that the entropy and SsLZ signatures we identify are intrinsic, sequence-encoded determinants of splicing fidelity rather than indirect tissue-specific covariates. Second, despite the strongly AT-rich genomes of all four species (GC content 22.6%–24.0%, Supplementary Table S2), RIs are still relatively enriched for the GC dinucleotide compared with CSIs. The fact that this enrichment is detectable against such a low GC background strengthens, rather than weakens, the parallel with the mammalian “IR code” described by Wong and colleagues (Wong et al., 2018; Wong and Schmitz, 2022), suggesting that GC dinucleotide enrichment in retained introns may extend beyond the high-GC mammalian context. Third, the inter-species variability in classifier performance, particularly the marked drop of Neural Networks on *T. malaccensis* (accuracy 0.709, MCC 0.419) compared with their good performance on *T. thermophila*, covaries with assembly quality (Pearson r ≈ −0.79 between the proportion of undetermined N bases in the assembly and the best-model accuracy across the four species, n = 4): *T. malaccensis* has the lowest N50 (496 kb), the lowest mean contig length (183 kb) and the highest N fraction (2.35%, Supplementary Table S2), suggesting that part of the divergence reflects assembly quality rather than purely biological divergence of splicing factors. We also note that *T. thermophila* received approximately 1.5× more sequencing reads (∼94 M paired vs. ∼62 M average for the other three species) and includes two conjugation samples (∼32% of its reads) absent in the other species (Supplementary Table S1); this greater biological and technical heterogeneity in the *T. thermophila* training set is one possible explanation for the slight performance edge of Adaboost M1 (accuracy 0.989) over the single C5.0 tree (accuracy 0.961) on this species, while C5.0 remained the best classifier on the other three. Fourth, the entropy thresholds identified by C5.0 (Shannon entropy <1.53 for CSIs vs. > 1.67 for RIs against a theoretical maximum H_max_ = log_2_ (4) = 2) span only ∼7% of the entropy range, indicating that even modest deviations from sequence repetitiveness are sufficient to bias the splicing decision. Notably, the genome-wide background nucleotide entropy of *T. thermophila*, computed from Supplementary Table S2 base counts, is H_bg_ ≈ 1.77 bits: the CSI threshold (1.53) therefore lies meaningfully below the bulk-genome composition, while RIs (≥1.67) approach the genome background. Read jointly, the two thresholds suggest that CSI introns are compositionally more biased than the average genome, consistent with selective pressure to maintain a recognisable splicing-signal signature, whereas RIs drift toward the bulk composition, indicating loss of compositional constraint. Finally, two limitations should be acknowledged: (i) under our convention CSIs are the positive (majority) class, so the metric most informative of the model’s actual utility is the recall on the minority RI class (i.e., specificity in Table 1), and (ii) the predicted RIs have not yet been validated experimentally: wet-lab confirmation by RT-PCR or targeted long-read sequencing of a subset of high-confidence predictions remains an obvious next step. The Galaxy and Docker implementations released here are designed to make such follow-up, and the extension of the framework to other tissue-poor unicellular eukaryotes (*Saccharomyces*, Plasmodium, Paramecium), straightforward. We emphasise that the present study is a foundational, single-genus benchmark; the framework is currently being extended to *Paramecium* and, subsequently, to non-ciliate tissue-poor eukaryotes such as *Saccharomyces* and *Plasmodium*, to assess generalizability across phyla and to characterise inter-phylum differences. Future work should also explore (i) leave-one-condition-out cross-validation to disentangle biological from sampling-related effects, and (ii) stratification of the xML rules by intron length class, since *Tetrahymena* introns are known to follow a bimodal length distribution corresponding to two splice-site recognition modes (Csűrös et al., 2016). A further direction is a systematic, tree-by-tree comparison of the species-specific decision rules, to test whether the same features and thresholds recur across the four *Tetrahymena* species.

## Data Availability

Sequence data analysed in this study are publicly available third-party datasets; no new sequence data were generated. RNA-seq runs are available at the European Nucleotide Archive under the study accessions SRP017606 (Tetrahymena thermophila; runs SRR636695–SRR636700), PRJNA167916/SRP013637 (T. borealis; SRR505872, SRR505873, SRR536858, SRR536859), PRJNA167917/SRP013638 (T. elliotti; SRR505874, SRR505875, SRR536842, SRR536843) and PRJNA167918/SRP013639 (T. malaccensis; SRR505878, SRR505879, SRR536826, SRR536827); the individual run accessions are listed in Supplementary Table S1. Genome assemblies are available from the Tetrahymena Genome Database (https://tet.ciliate.org/downloads.php). The Galaxy workflows, custom scripts and trained models supporting the analysis are available at https://zenodo.org/records/21740113, [10.5281/zenodo.21740113]. The Docker container reproducing the analytical environment is available at https://hub.docker.com/r/dockervito/galaxybiounicam4.
