# Supplementary material for: Entropy-driven machine learning for deciphering mRNA rearrangement by splicing in complex microorganisms
Source: Front Bioinform. 2026 Sep 17;6:1889469. doi: 10.3389/fbinf.2026.1889469 (PMC13627988; doi:10.3389/fbinf.2026.1889469)
Supplement: Supplementary file 1 [file Supplementaryfile1.docx]

Supplementary Material

# 1 Supplementary Methods

## 1.1 Features description: after the extraction of the Retained Introns (RIs) and the Constitutively Spliced Introns (CSIs) nucleotide sequences, various numerical and categorical features were selected to develop supervised classification models to distinguish between the two classes.

**1.1.1-Probabilities of Six Pair Nucleotides in the primary structures** (Karlin and Burge, 1995). It considers the k-combinations (*k* = 2) of the nucleotide set *N* = *{A, C, G, T}* without repetition and without order, represented by the six pairs *AG,AC,AT,GC,GT* and *CT*, found within the sequence. For instance, the probability for the pairs *GC* (or *CG*) is:

$$\hat{\varphi}_{GC} = \frac{1}{c_{k}}\sum_{k=1}^{c_{k}} \left\| P^{G} - P^{C} \right\|.$$

**1.1.2- Entropy based features** estimated both for primary and secondary structures. Entropy quantifies the order or disorder of a symbolic sequence. In this context, a sequence *S* composed of *n* symbols *s_k_* (where, k = 0, 1, . . . , *n*) drawn from an alphabet α of size *p* is classified as disordered when all symbols and their combinations occur with equal probability. Conversely, a sequence is deemed ordered if its symbols *s_k_*, or certain subsequences, recur repeatedly as patterns or repetitive motifs within the sequence. The measure of order or disorder within a sequence is quantified using Shannon entropy (base-2 logarithm yields entropy in bits, consistent with H_max = log₂(4) = 2 used in the main manuscript)

$H = -\sum_{k=1}^{p} \varphi_{k}\log\left( \varphi_{k} \right)$ (1),

which quantifies the informational content of a sequence. To define Shannon entropy (1), consider a categorical random variable with an alphabet of size *p* and corresponding cell probabilities *ϕ1, ϕ2, . . . , ϕp* where *ϕk ≫ 0*, and $\sum_{k} \varphi_{k} = 1$. These probabilities must be estimated. A widely used estimator of entropy is the maximum likelihood estimator, where *yk* is the count of particular character k ∈ A in the given sequence, given by

$n = \sum_{p} y_{k}.$.

The formula (1) can be rewritten by $H^{ML} = -\sum_{k=1}^{p} \varphi_{k}^{ML}\log\left( \varphi_{k}^{ML} \right)$, where

$\varphi^{ML} = \frac{y_{k}}{n}$. When n ≫ p, meaning the alphabet size is small relative to the number of observations, entropy can be reliably inferred. In such scenarios, it is well-established that the maximum likelihood estimator provides optimal performance. We note that iREAD also uses a Shannon-entropy-based criterion to call IR events, but that criterion is computed on read coverage uniformity rather than on the nucleotide sequence; the entropy feature defined here is therefore mathematically independent of the IR-calling criterion.

**1.1.3- Lempel–Ziv Complexity Measure** estimated both for primary and secondary structures. LZ has been used for complexity characterization of DNA sequences (Gusev et al., 1999) and to develop new methods for discovering patterns in DNA sequences (Orlov and Potapov, 2004). This complexity measure is related to the number of distinct substrings (i.e., patterns) and the rate of their occurrence along a given sequence (Gusev et al., 1999). Each time a new pattern is encountered, the complexity count increases by one. The final LZ complexity score reflects how many distinct patterns were found, and thus measures the repetitiveness of an RNA sequence. Note that the standard Lempel–Ziv complexity scales with sequence length; if a difference in mean length exists between RIs and CSIs, a length-normalised variant (Zhang Y, Hao P. J Math Chem. 2009;46(4):1203-12, doi:10.1007/s10910-008-9512-2) should be used to disentangle the contributions of repetitiveness and length.

**1.1.4- Secondary Structure Accessibility and Effective Distance features.** Given a secondary structure *S = s1, . . . , sn,* where *s ∈ {(, ., )},* we apply the effective distance and accessibility measures to the intron subsequence *Ŝ = S[i, n]*. Here, *Ŝ* represents the sequence segment in the range between *i=*branch point (BP) and the 3’ss, obtained by excluding all bases preceding index *i* that denotes the starting position of BP (see Figure S3). The linear distance is defined as the number of nucleotides (nt) between the BP and the 3’ss. The effective distance is the counts of . (dot), i.e. unpaired nucleotides, starting from the beginning to the end of *Ŝ*. This measure can help to understand how these sites (BP and 3’ss) are “effectively” close to each other in the folded RNA, rather than in the “linear” structure.

The accessibility measure counts how much the signal is more or less hidden in the mRNA structure. The overlap of the secondary structure to cis-regulatory elements (Wang and Burge, 2008) can hinder their recognition by proteins and RNAs in recognizing a splice site. Accessibility estimates how exposed a splice signal is within the folded RNA: an accessible signal can be bound by protein factors, whereas a signal sequestered in secondary structure is less likely to be recognised. The approach introduced by Plass and Eyras (2014) was used to calculate the average accessibility. In addition, splice site strength was quantified using MaxEntScan, providing a numerical estimate of 5’ and 3’ splice site quality, which was included among the predictive features.

**1.1.5- Feature set summary and data quality validation.** The complete feature set used for classification consists of: (i) six dinucleotide pair probabilities (AG, AC, AT, GC, GT, CT) computed on the primary intronic sequence (§1.1.1); (ii) Shannon entropy on the primary sequence and on the dot-bracket secondary structure (§1.1.2); (iii) Lempel–Ziv complexity on the primary sequence and on the dot-bracket secondary structure (§1.1.3); (iv) effective BP–to–3′-splice-site distance and average accessibility, the linear branch-point-to-3′-splice-site distance, and the numbers of internal loops and stem-loops, computed on the secondary structure (§1.1.4); (v) MaxEntScan-derived 5′ and 3′ splice-site strength scores. After removal of correlated variables and PCA, a subset of these features was retained as input for the four classifiers compared in Table 1 of the main manuscript; the features actually used by the C5.0 model are those shown in Figures 2, 3 and 5, whose abbreviations are defined in Table S4. Some candidate features (the MaxEntScan scores and the secondary-structure Shannon entropy) were not retained after this selection and therefore do not appear in the figures. As a quality-control validation, all four genome assemblies (Table S2) satisfy Chargaff’s second parity rule, with |numA − numT|/numA < 0.4% and |numG − numC|/numG < 0.4% in every species, ruling out strand-specific compositional bias as a confounder for the feature analyses presented here.

# 2 Supplementary Figures and Tables

## 2.1 Supplementary Figures

Fig. S1 Traditional classification of basic types of alternative RNA splicing events. Exons are represented as blocks, introns as lines in between.

Fig. S2. Workflow pipeline for studying IR in Tetrahymena species. The pipeline includes five main

phases: A Conceptualization: Apply ML to investigate IR phenomena; B Data Acquisition: Extract intronic sequences from the Tetrahymena genome after comparison with various transcriptomes, preprocessed to remove low-quality reads; C Data Preparation: A bioinformatics pipeline for data cleaning, mapping, transcriptome assembly, isoform identification, extraction of retained introns (RIs) and constitutively spliced introns (CSIs), and feature engineering; D Machine Learning Modeling: ML pipeline for classification, including PCA, data balancing, dataset splitting, cross-validation, and model evaluation; E Results Interpretation: Analyze results using explainable machine learning (xML; Vimbi et al., 2024) approaches.

Fig. S3 Branch Point (BP) position and Effective Distance in the RNA Secondary Structure Elements.

Fig. S4 Adopted machine learning pipeline.

Fig. S5 BioUnicam Utilities: Galaxy iREAD workflow visual specification.

Supplementary Tables

Table S1: Transcriptomic Data used in this study

| **Organism** | **ID** | **Condition** | **Sequencing platform** | **Number of reads** | **Sequencing types** | **Percentage of aligned mapping (%)** |
| --- | --- | --- | --- | --- | --- | --- |
| *Tetrahymena thermophila* | SRR636695 | Growth-m | Illumina Analyzer | 30812705 | Paired | 96.75 |
|  | SRR636696 | Starvation-3h-VI | Illumina Analyzer | 15978436 | Paired | 97.37 |
|  | SRR636697 | Starvation-3h-V | Illumina Analyzer | 8939127 | Paired | 90.39 |
|  | SRR636698 | Starvation-15h-VI | Illumina Analyzer | 8254521 | Paired | 85.35 |
|  | SRR636699 | Conjugation-2h | Illumina Analyzer | 16329020 | Paired | 97.05 |
|  | SRR636700 | Conjugation-8h | Illumina Analyzer | 13692298 | Paired | 92.19 |
| *Tetrahymena borealis* | SRR536859 | Growth | Illumina HiSeq 2000 | 14915466 | Paired | 90.20 |
|  | SRR536858 | Starvation | Illumina HiSeq 2000 | 16292687 | Paired | 90.35 |
|  | SRR505873 | Growth | Illumina HiSeq 2000 | 14923292 | Paired | 91.02 |
|  | SRR505872 | Starvation | Illumina HiSeq 2000 | 15857549 | Paired | 90.13 |
| *Tetrahymena elliotti* | SRR536843 | Starvation | Illumina HiSeq 2000 | 16317662 | Paired | 90.89 |
|  | SRR536842 | Growth | Illumina HiSeq 2000 | 14946584 | Paired | 90.14 |
|  | SRR505875 | Starvation | Illumina HiSeq 2000 | 15798072 | Paired | 91.25 |
|  | SRR505874 | Growth | Illumina HiSeq 2000 | 14486996 | Paired | 90.26 |
| *Tetrahymena malaccensis* | SRR505878 | Growth | Illumina HiSeq 2000 | 14873192 | Paired | 90.85 |
|  | SRR505879 | Starvation | Illumina HiSeq 2000 | 16801891 | Paired | 89.69 |
|  | SRR536826 | Growth | Illumina HiSeq 2000 | 15072192 | Paired | 90.22 |
|  | SRR536827 | Starvation | Illumina HiSeq 2000 | 16718449 | Paired | 90.54 |

| Table S2. Transcriptomes statistic details after the RNA STAR mapping. GC content: percentage of G and C bases over the total; L50: minimum number of contigs/scaffolds that together represent at least 50% of the total length; len N50: length of the shortest contig/scaffold among those that, ordered by length, cumulatively reach 50% of the total length; len min: length of the shortest contig; len mean: average contig length; len median: median contig length; len max: length of the longest contig; num A / C / G / T: number of adenine, cytosine, guanine and thymine nucleotides; num N: number of “N” bases (undetermined positions); num bp: total number of bases (including N); num bp not N: total number of bases excluding N; num seq: total number of contigs/scaffolds. Note that *T. elliotti* displays a markedly skewed contig length distribution (mean/median ≈ 47×) compared with the other three species (1.5–2.5×), reflecting a few very long contigs alongside many smaller ones; this should be considered when interpreting cross-species comparisons. | | | | |
| --- | --- | --- | --- | --- |
|  | *T. thermophila* | *T. elliotti* | *T. borealis* | *T. malaccensis* |
| GC content (%) | 22.7 | 22.9 | 24.0 | 22.6 |
| L50 | 32 | 27 | 44 | 64 |
| len N50 | 829795 | 1022547 | 638740 | 496146 |
| len min | 21260 | 1007 | 1036 | 1028 |
| len mean | 570917 | 274433 | 287711 | 182548 |
| len median | 387027 | 5784 | 130346 | 72737 |
| len max | 3329751 | 3762730 | 3433454 | 2173071 |
| num A | 40173351 | 34207751 | 35027702 | 40307994 |
| num C | 11504615 | 10156034 | 11060093 | 11771935 |
| num G | 11543842 | 10189089 | 11040528 | 11741544 |
| num N | 2013963 | 2162242 | 1375227 | 2504422 |
| num T | 40123954 | 34122530 | 35002526 | 40345976 |
| num bp | 103345795 | 90837646 | 93506076 | 106671871 |
| num bp not N | 101331832 | 88675404 | 92130849 | 104167449 |
| num seq | 181 | 331 | 325 | 584 |

Table S3: tools used to detect intron retention

| **Tool** | **Description** | **Website** |
| --- | --- | --- |
| IRFinder | Detecting IR from RNA-Seq experiments | github.com/williamritchie/IRFinder |
| Kma | R package for IR detection | github.com/pachterlab/kma |
| spliceR | AS identification/quantification | bioconductor.org/packages/spliceR |
| ASTALAVISTA | AS quantification and analysis | astalavista.sammeth.net |
| iREAD | intron REtention Analysis and Detector | https://github.com/genemine/iread |

Table S4: Abbreviations of the feature names used in the figures (Figs. 2, 3 and 5).

| **Abbreviation** | **Feature** | **Definition** |
| --- | --- | --- |
| PhiAU, PhiGC, PhiGU | Dinucleotide-pair probability | Probability of the unordered nucleotide pair in the primary sequence (RNA notation, U = T): PhiAU = φ(A,T), PhiGU = φ(G,T), PhiGC = φ(G,C); §1.1.1 |
| Shannon En. | Shannon entropy (primary sequence) | Shannon entropy of the primary nucleotide sequence; §1.1.2 |
| LZ | Lempel–Ziv complexity (primary) | Lempel–Ziv complexity of the primary sequence; §1.1.3 |
| SsLZ | Lempel–Ziv complexity (secondary) | Lempel–Ziv complexity of the dot-bracket secondary structure; §1.1.3 |
| Eff.Distance | Effective BP–3′SS distance | Number of unpaired nucleotides between the branch point and the 3′ splice site in the folded structure; §1.1.4 |
| AA | Average accessibility | Average accessibility of the secondary structure; §1.1.4 |
| BP | Linear BP–3′SS distance | Linear distance (nt) between the branch point and the 3′ splice site; §1.1.4 |
| Int.Loop | Internal loops | Number of internal loops in the secondary structure |
| Stem-loop | Stem-loops | Number of stem-loops in the secondary structure |
| SL | Sequence length | Intron length (nt); excluded from the model (Fig. 5) |
